# Efficacy and Safety of Duloxetine in Patients with Chronic Low Back Pain Who Used versus Did Not Use Concomitant Nonsteroidal Anti-Inflammatory Drugs or Acetaminophen: A Post Hoc Pooled Analysis of 2 Randomized, Placebo-Controlled Trials

**DOI:** 10.1155/2012/296710

**Published:** 2012-03-18

**Authors:** Vladimir Skljarevski, Peng Liu, Shuyu Zhang, Jonna Ahl, James M. Martinez

**Affiliations:** ^1^Lilly Research Laboratories, Eli Lilly and Company, Drop Code 1542, Indianapolis, IN 46285, USA; ^2^Lilly USA, LLC., Drop Code 4133, Indianapolis, IN 46285, USA

## Abstract

This subgroup analysis assessed the efficacy of duloxetine in patients with chronic low back pain (CLBP) who did or did not use concomitant nonsteroidal anti-inflammatory drugs (NSAIDs) or acetaminophen (APAP). Data were pooled from two 13-week randomized trials in patients with CLBP who were stratified according to NSAID/APAP use at baseline: duloxetine NSAID/APAP user (*n* = 137), placebo NSAID/APAP user (*n* = 82), duloxetine NSAID/APAP nonuser (*n* = 206), and placebo NSAID/APAP nonuser (*n* = 156). NSAID/APAP users were those patients who took NSAID/APAP for at least 14 days per month during 3 months prior to study entry. An analysis of covariance model that included therapy, study, baseline NSAID/APAP use (yes/no), and therapy-by-NSAID/APAP subgroup interaction was used to assess the efficacy. The treatment-by-NSAID/APAP use interaction was not statistically significant (*P* = 0.31) suggesting no substantial evidence of differential efficacy for duloxetine over placebo on pain reduction or improvement in physical function between concomitant NSAID/APAP users and non-users.

## 1. Introduction

Low back pain has a lifetime prevalence rate of 80% in the United States and is one of the primary causes of disability in individuals younger than 45 years of age [1, 2]. Low back pain usually resolves spontaneously within a few days or weeks, but for some individuals, this pain becomes chronic [1]. Commonly prescribed medications for chronic low back pain (CLBP) include nonsteroidal anti-inflammatory drugs (NSAIDs), opioids, muscle relaxants, anticonvulsants, and tricyclic antidepressants (TCAs) [3]. Over-the-counter medications that are frequently used include acetaminophen (APAP), aspirin, and certain NSAIDs [4]. However, there is no clinical evidence to support the efficacy of any of these agents in CLBP [4, 5]. Furthermore, a number of these treatments pose safety risks that include sedation, respiratory depression and addiction (opioids), gastrointestinal bleeding and ulcers, and cardiovascular events (NSAIDs) [6]. In addition, antidepressants with serotonin reuptake inhibition properties may increase the risk of bleeding events [7, 8], either when taken alone or in combination with other drugs that affect coagulation, such as NSAIDs [9].

Duloxetine hydrochloride (hereafter referred to as duloxetine) is a potent serotonin and norepinephrine reuptake inhibitor (SNRI) that has been approved by the United States Food and Drug Administration for the management of diabetic peripheral neuropathic pain, fibromyalgia, and chronic musculoskeletal pain (as established in studies in CLBP and chronic pain due to osteoarthritis). It has also been approved for the treatment of major depressive disorder (MDD) and generalized anxiety disorder (GAD) [10].

In two 13-week trials of duloxetine versus placebo in patients with CLBP, one trial [11] reported significantly greater pain reduction with duloxetine treatment at endpoint; whereas the other reported significant separation from placebo at weeks 3–11, but superiority was not demonstrated at endpoint [12]. Because these trials allowed concomitant use of NSAIDs or APAP if patients used these analgesics regularly prior to study entry, subgroup analyses were conducted to assess whether or not concomitant use of the allowed analgesics had an effect on the efficacy of duloxetine. The results of the subgroup analyses were not significant for either trial, but were limited by sample size. To increase the statistical power and to better understand the advantage of duloxetine over placebo between the groups of patients who concomitantly used these analgesics and those who did not, we conducted a post hoc analysis of data pooled from these two studies. The safety of duloxetine with concomitant use of these analgesics was also evaluated.

## 2. Materials and Methods

This was a post hoc analysis of data pooled from two 13-week, multicenter, randomized, double-blind trials of the efficacy of duloxetine (doses of 60 QD and 120 QD were pooled for this analysis) compared with placebo on the reduction of average pain severity, improvement in physical function, and in patient global impression of improvement [11, 12]. Both studies were compliant with International Conference on Harmonization guidelines on good clinical practices, and each protocol was approved by the Ethical Review Board for each site. All patients provided written informed consent before beginning any study procedures.

Patients included in these studies were outpatients who were at least 18 years of age with a clinical diagnosis of CLBP; with pain restricted to the lower back (Class 1) or associated with radiation to the proximal portion of the lower limb only (Class 2) according to the Quebec Task Force (QTF) on Spinal Disorders [13]; with pain present on most days for ≥6 months, and weekly average pain severity ratings ≥4 (on a 0–10 numerical scale) during the week prior to randomization. Exclusion criteria included clinical or radiographic evidence of radicular compression or spinal stenosis, presence of spondylolisthesis grade 3–4, history of ≥1 low-back surgery, any low-back surgery within 12 months, or invasive procedures to reduce low-back pain within 1 month. Patients with MDD, body mass index >40, or seeking disability compensation related to back pain were excluded.

At baseline, patients were stratified according to concomitant NSAID and/or APAP use status prior to study entry and were randomized to duloxetine and placebo within each stratum. Users were defined as those patients answered “yes” to a question soliciting whether or not they were taking a therapeutic dose of NSAID and/or APAP for ≥14 days per month for 3 months immediately preceding the study. Because the use of these analgesics was recorded as a global “yes” response, this stratum was referred to as the NSAID/APAP user group. Patients who were NSAID/APAP users were allowed to continue with their stable regimen of NSAID/APAP throughout the entire study.

For this analysis, efficacy measures included the Brief Pain Inventory (BPI) [14] 24-hour average pain severity item (referred to hereafter as BPI average pain severity) (range, 0 = no pain to 10 = pain as bad as you can imagine); the Roland-Morris Disability Questionnaire (RMDQ-24) [15] (scale range, 0 = no disability to 24 = severe disability); the Patient Global Impression of Improvement (PGI-I) [16]. The PGI-I is a scale on which patients provide ratings of their overall impression of how they are feeling since treatment began with the following range of choices from 1 = very much better to 4 = no change to 7 = very much worse. Response to treatment was defined as at least a 50% decrease from baseline in BPI average pain severity.

To assess and compare the efficacy of duloxetine over placebo between NSAID/APAP use subgroups, we utilized an analysis of covariance (ANCOVA) model to estimate least-squares mean changes from baseline to endpoint in BPI average pain severity ratings and RMDQ-24 scores. The ANCOVA model included a fixed continuous covariate of baseline value, fixed categorical effects of therapy (duloxetine or placebo), study, NSAID/APAP use (yes/no), and therapy-by-NSAID/APAP subgroup interaction. Response at endpoint was also analyzed using a logistic regression model with terms for therapy, study, NSAID/APAP use (yes/no), and therapy-by-NSAID/APAP subgroup interaction. Statistically significant difference in duloxetine efficacy between subgroups for reduction in BPI average pain severity, improvement in RMDQ-24 scores, and BPI pain response was determined by a therapy-by-NSAID/APAP subgroup interaction that was *P* < .1. The number and proportion of patients who reached a PGI-I rating of 1 or 2 at endpoint were summarized by treatment and by NSAID/APAP use subgroup. Subsequent treatment odds ratios were calculated for each subgroup, then compared between subgroups with the Breslow-Day test, and statistical significance was noted at *P* < .1. For patients with missing outcomes (due to early dropout), the last nonmissing observation was treated as their endpoint value in the analyses.

Safety assessments included discontinuation due to adverse events (AEs), the most common treatment-emergent adverse events (TEAEs), and those possibly related to NSAID/APAP use (bleeding and cardiovascular events). Incidence rates were compared between treatment groups using Cochran-Mantel-Haenszel test controlling for study, and statistical significance was noted at *P* < .05.

## 3. Results

### 3.1. Patient Disposition

There was no significant between-treatment difference in rates of study completion in either NSAID/APAP use subgroup (Table 1). There was a higher percentage of duloxetine-treated patients versus placebo, who discontinued due to AEs in the NSAID/APAP nonuser subgroup (*P* = .002). For any of the other reasons leading to discontinuation, there were no significant between-treatment differences in either NSAID/APAP use subgroup.

### 3.2. Demographics and Clinical Characteristics

Patients were stratified according to NSAID/APAP use at baseline: duloxetine NSAID/APAP user (*n* = 137), placebo NSAID/APAP user (*n* = 82), duloxetine NSAID/APAP nonuser (*n* = 206), and placebo NSAID/APAP nonuser (*n* = 156). Patient demographics and clinical characteristics are summarized in Table 2. Most of the patients were female, Caucasian, and in their early fifties. Most had pain restricted to lower back (Class 1 per QFT on spinal disorders), with an average duration of CLBP since onset of at least 9 years. At baseline, the mean BPI average pain severity rating was 6, and the mean RMDQ-24 rating for physical function was about 9.5.

## 4. Efficacy

**Figure 1:**
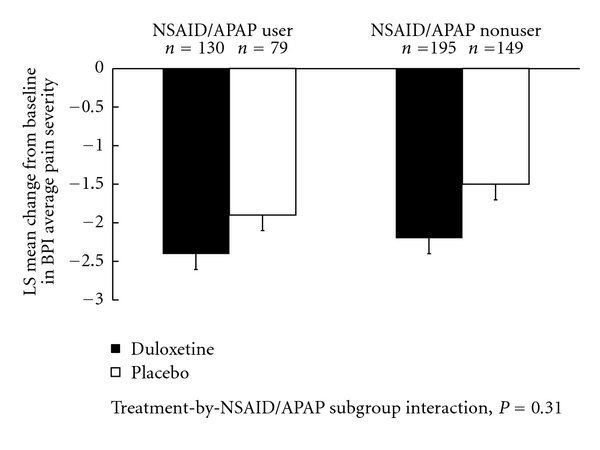
Estimated least-squares (LS) mean changes from baseline and standard errors in BPI average pain severity in patients who concomitantly used or did not use NSAID or APAP.

**Figure 2:**
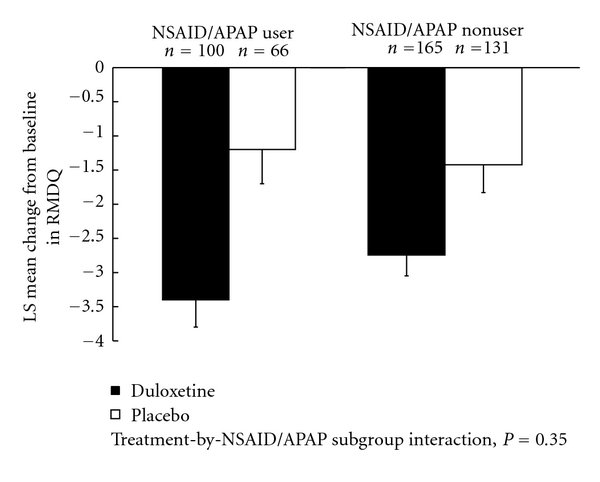
Estimated least-squares (LS) mean changes from baseline and standard errors in RMDQ rating in patients who concomitantly used or did not use NSAID or APAP.

**Figure 3:**
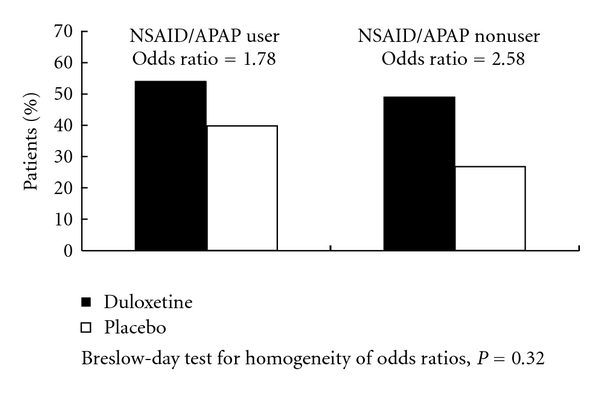
Percentage of patients who felt “much better” or “very much better” at endpoint.

Mean changes from baseline in efficacy measures are shown in Figures 1 and 2. Treatment-by-NSAID/APAP use subgroup interactions were not significant for reduction n BPI average pain severity (*P* = .31, Figure 1), or for improvement in physical function assessed by the RMDQ-24 (*P* = .35, Figure 2). These results suggest that there was no substantial evidence of differential duloxetine efficacy on pain reduction or improvement in physical function between concomitant NSAID/APAP users and nonusers. The frequency of PGI-I responses that were “much better” or “very much better” (PGI-I endpoint score ≤2) is presented in Figure 3. In both NSAID/APAP use subgroups, a higher percentage of duloxetine-treated patients achieved PGI-I ≤2 at endpoint, but the treatment odds ratios were not significantly different between the two subgroups (*P* = 0.32). Therefore, significant differential treatment effects of duloxetine on PGI-I were not observed between concomitant NSAID/APAP users and nonusers.

The criterion for achieving a pain response was met by a higher percentage of duloxetine-treated patients in both NSAID/APAP use subgroups (46.2% of users, and 43.6% of nonusers) than patients treated with placebo (38.0% of users, and 27.5% of nonusers). The treatment-by-NSAID/APAP use subgroup interaction was not significant (*P* = 0.28), which suggests that the duloxetine treatment effects on achieving a pain response were not statistically significantly different between concomitant NSAID/APAP users and nonusers.

## 5. Safety

The most common AEs that lead to discontinuation in the NSAID/APAP use subgroup in duloxetine- treated patients were erectile dysfunction and nausea (both events, *n* = 2, 1.5%); events in the nonuser subgroup included nausea, insomnia and somnolence (each event, *n* = 3, 1.5%).

TEAEs within NSAID/APAP use subgroups that occurred at a rate of at least 5% with duloxetine treatment and were significantly more frequent than with placebo are summarized in Table 3. Among NSAID/APAP users, the frequency of nausea, dry mouth, constipation, somnolence, and fatigue were significantly greater in patients who received duloxetine versus placebo. In addition to these TEAEs, insomnia, and dizziness were significantly more frequent in patients who received duloxetine versus placebo among the NSAID/APAP nonusers. Cardiovascular and bleeding-related TEAEs are summarized in Table 4. In either NSAID/APAP use subgroup, between-treatment differences in the frequency of these events were not significant.

## 6. Discussion

There are few published CLBP studies with nonopioid analgesics that allowed concomitant NSAID/APAP use. Two studies investigated the efficacy of TCAs for pain reduction in patients who were allowed to continue taking NSAIDS. One of those two evaluated nortriptyline against placebo [17] and the other compared maprotiline with paroxetine [18], but neither study reported efficacy outcome comparisons between NSAID/APAP users and nonusers. Another CLBP study examined the efficacy of pregabalin combined with celecoxib, and the results suggested that combination treatment was more efficacious than treatment with either medication alone [19].

The post hoc analysis reported here included two clinical trials of duloxetine that allowed concomitant NSAID/APAP for those patients who regularly used them prior to study entry. The use of additional analgesics in a pain trial is associated with the risk of reduced assay sensitivity, and possibly a high placebo response [17]. This was observed in one of the two duloxetine CLBP trials [12] and in the NSAID/APAP use subgroup in this analysis. However, the treatment-by-NSAID/APAP use subgroup interaction was not significant in the analyses of various efficacy measures, which suggests that the advantages of duloxetine over placebo in pain reduction and improvement in function were not significantly different between subgroups.

The safety profile with regards to TEAEs in either NSAID/APAP use subgroup did not differ from those reported previously in duloxetine trials. Although the occurrence of bleeding-related and cardiac-related events noted in this post hoc analysis was low in both NSAID/APAP use group, caution is warranted for concomitant use of NSAID/APAP with duloxetine. This precautionary statement is based upon observations that medications that act to inhibit serotonin reuptake may be associated with an increased risk of bleeding events [9], and the use of these drugs in combination with medications that affect coagulation, including NSAIDs, may increase this risk.

This study is limited by the lack of complete information regarding dosing and frequency of concomitant NSAID or APAP use. In addition, any NSAID or APAP use less than 14 days/month would have classified patients as nonusers, which also included patients that did not use these analgesics at all, and patients who used them sporadically. In addition, users were identified at baseline by responding “yes” to a questionnaire regarding the use of either NSAIDs or APAP, instead of regarding the use of one or the other or both, so this lack of information limited further analysis. Also, the studies included in these analyses were not powered to detect differential treatment effect between NSAID/APAP use subgroups. Therefore, the comparisons between NSAID/APAP use subgroups in this study should be viewed in that light. In addition, the sample size and the short duration of the studies also limited the occurrence and detection of rare bleeding events. Finally, these studies excluded individuals with certain comorbidities, so these results may not extend to all individuals in the general population who present with CLBP.

## 7. Conclusions

In this post hoc analysis of data pooled from two studies in patients with CLBP, there were no statistically significant differences in the treatment advantage of duloxetine over placebo on measures of pain reduction, improved physical function, or patient global impression of improvement observed between concomitant NSAID/APAP users and nonusers. In other words, concomitant use of an NSAID or APAP did not significantly enhance or interfere with the efficacy of duloxetine. The safety of duloxetine with concomitant NSAID/APAP use was consistent with the known duloxetine safety and tolerability profile.

## Figures and Tables

**Table 1:** Patient disposition.

	NSAID/APAP user			NSAID/APAP nonuser		
	Duloxetine *N* = 137 *n* (%)	Placebo *N* = 82 *n* (%)	*P* value	Duloxetine *N* = 206 *n* (%)	Placebo *N* = 156 *n* (%)	*P* value
Completed study	90 (65.7)	63 (76.8)	.10	136 (66.0)	117 (75.0)	.08
Reason for discontinuation:						
Adverse event	21 (15.3)	5 (6.1)	.051	39 (18.9)	12 (7.7)	.002
Lack of efficacy	7 (5.1)	4 (4.9)	1.00	5 (2.4)	7 (4.5)	.38
Lost to follow up	5 (3.6)	0	.16	7 (3.4)	3 (1.9)	.53
Protocol violation	5 (3.6)	4 (4.9)	.73	5 (2.4)	2 (1.3)	.70

Abbreviation: APAP, acetaminophen; NSAID, nonsteroidal anti-inflammatory drug.

**Table 2:** Baseline characteristics.

	NSAID/APAP user		NSAID/APAP nonuser	
	Duloxetine *N* = 137	Placebo *N* = 82	Duloxetine *N* = 206	Placebo *N* = 156
Age in years, mean (SD)	53.1 (14.7)	51.4 (13.3)	53.5 (14.9)	53.1 (13.6)
Female, *n* (%)	89 (65.0)	52 (63.4)	114 (55.3)	85 (54.5)
Ethnicity, *n* (%)				
African American	3 (2.2)	3 (3.7)	19 (9.2)	13 (8.3)
Caucasian	108 (78.8)	61 (74.4)	160 (77.7)	123 (78.9)
East Asian	0	0	2 (1.0)	3 (1.9)
Hispanic	25 (18.3)	17 (20.7)	22 (10.7)	15 (9.6)
Native American	0	0	1 (0.5)	2 (1.3)
West Asian	1 (0.7)	1 (1.2)	2 (1.0)	0
CLBP duration since onset, years, mean (SD)	11.4 (11.5)	9.2 (9.1)	10.8 (11.1)	10.3 (9.0)
QT F class 1, *n* (%)	94 (72.9)	63 (79.8)	150 (75.4)	99 (68.3)
BPI average pain, mean (SD)	6.1 (1.6)	6.0 (1.6)	5.9 (1.6)	6.1 (1.7)
RMDQ-24, mean (SD)	9.6 (5.0)	8.6 (4.8)	9.1 (4.5)	9.8 (5.3)

There was a statistically significant difference in RMDQ-24 scores between treatments in the nonuser subgroup, but this difference was not considered clinically significant.

Abbreviations: APAP, acetaminophen; BPI, Brief Pain Inventory; CLBP, chronic low back pain; NSAID, nonsteroidal anti-inflammatory drugs; QTF, Quebec Task Force; RMDQ-24, Roland-Morris Disability Questionnaire, SD, standard deviation.

**Table 3:** Treatment-emergent adverse events within either NSAID/APAP use subgroups that occurred at a rate of at least 5% with duloxetine treatment and were significantly more frequent than with placebo.

	NSAID/APAP User			NSAID/APAP Nonuser		
	Duloxetine *N* = 137 *n* (%)	Placebo *N* = 82 *n* (%)	*P* value	Duloxetine *N* = 206 *n* (%)	Placebo *N* = 156 *n* (%)	*P* value
At least 1 adverse event	92 (67.2)	51 (62.2)	.850	141 (68.5)	77 (49.4)	<.001
Nausea	25 (18.3)	3 (3.7)	.006	26 (12.6)	4 (2.6)	<.001
Dry mouth	14 (10.2)	1 (1.2)	.018	21 (10.2)	4 (2.6)	.004
Constipation	13 (9.5)	1 (1.2)	.041	17 (8.3)	1 (0.6)	.001
Somnolence	13 (9.5)	0	.006	12 (5.8)	1 (0.6)	.012
Fatigue	10 (7.3)	0	.012	15 (7.3)	1 (0.6)	.003
Insomnia	13 (9.5)	4 (4.9)	.346	23 (11.2)	4 (2.6)	.004
Dizziness	10 (7.3)	2 (2.4)	.197	15 (7.3)	3 (1.9)	.023

*P* values from Cochran-Mantel-Haenszel test.

Abbreviation: APAP, acetaminophen; NSAID, nonsteroidal anti-inflammatory drugs.

**Table 4:** Bleeding-related or cardiac-related treatment-emergent adverse events.

	NSAID/APAP user			NSAID/APAP nonuser		
	Duloxetine *N* = 137 *n* (%)	Placebo *N* = 82 *n* (%)	*P* value	Duloxetine *N* = 206 *n* (%)	Placebo *N* = 156 *n* (%)	*P* value
*Bleeding-related *						
Tendency to bruise	1 (0.7)	0	.52	0	0	—
Eye hemorrhage	0	0	—	1 (0.5)	1 (0.6)	.70
Hemorrhagic cyst	0	0	—	1 (0.5)	0	.31
Rectal hemorrhage	0	0	—	1 (0.5)	0	.44
*Cardiac-related*						
Palpitations	5 (3.7)	0	.14	1 (0.5)	1 (0.6)	.86
Hypertension	3 (2.2)	1 (1.2)	.84	4 (1.9)	2 (1.3)	.76
Myocardial infarction	1 (0.7)	0	.52	0	1 (0.6)	.32
Tachycardia	1 (0.7)	0	.29	2 (1.0)	0	.15
Transient ischemic attack	1 (0.7)	0	.52	1 (0.5)	0	.31
Heart rate increased	0	0	—	1 (0.5)	0	.44
Hypertensive crisis	0	0	—	1 (0.5)	0	.44
Carotid artery stenosis	1 (0.7)	0	.52	0	0	—

Abbreviation: APAP, acetaminophen; NSAID, nonsteroidal anti-inflammatory drugs.
